# The Clinical Characteristics and Outcomes of Hemorrhagic Fever With Renal Syndrome in Pregnancy

**DOI:** 10.3389/fmed.2022.839224

**Published:** 2022-02-21

**Authors:** Danfeng Ren, Shan Fu, Taotao Yan, Tianzhi Ni, Ze Zhang, Mengmeng Zhang, Jingwen Zhou, Nan Yang, Yuan Yang, Yingli He, Tianyan Chen, Yingren Zhao, Jinfeng Liu

**Affiliations:** ^1^The First Affiliated Hospital of Xi'an Jiaotong University, Xi'an, China; ^2^Shaanxi Clinical Research Center of Infectious Diseases, Xi'an, China

**Keywords:** hemorrhagic fever with renal syndrome, pregnancy, Hantaan virus, maternal outcomes, fetal outcomes

## Abstract

Pregnant women with hemorrhagic fever with renal syndrome (HFRS) are a significant challenge for clinicians. The clinical characteristics of HFRS in pregnant women and its influence on both the pregnant women and fetus have yet to be clarified clearly. To highlight the specific clinical features of HFRS in pregnant women and the outcomes of pregnant women with HFRS and their fetuses, we screened pregnant women with HFRS from inception to May 1^st^ 2021. We also conducted a comparison with non-pregnant women complicated with HFRS. Twenty-seven pregnant women and 87 non-pregnant women with complete electronic medical records were enrolled for final analyses; 55.6% (15/27) and 21.8% (19/87) were diagnosed as critical type in pregnant women and non-pregnant women, respectively. Compared with non-pregnant patients, there was a significantly higher likelihood of critical status in pregnant patients; the risk was significantly higher in late trimester (*p* <0.001). In addition, hypoalbuminemia and anemia were also evident in pregnant patients (*p* = 0.04, *p* <0.001, respectively). Leukocyte count, especially when higher than 15 × 10^9^/L, was significantly correlated with disease severity (*p* = 0.009). After comprehensive therapy, 26 pregnant patients recovered without sequelae. Five fetal adverse events were reported during hospitalization. All adverse events were observed in mothers with critical types (*p* = 0.047, X_2_ = 4.909) and occurred in the later trimester. Collectively, our data show that pregnant woman with HFRS during the third trimester presents a more severe condition, especially those with leukocytosis. However, the majority of those pregnant patients could recover with comprehensive treatment and undergo normal labor.

## Introduction

Hantavirus infection, which can cause hemorrhagic fever with renal syndrome (HFRS) and hantavirus pulmonary syndrome (HPS), remains a worldwide public health problem (1). An estimated 20,000 patients are reported each year globally, the majority in Europe, America, and Asia (2). China is the most seriously affected country and accounts for over 90% of all HFRS cases (3, 4). High mortality rates, of up to 12% in Asia and Europe, and up to 40% in the Americas, have been reported; the mortality rate

depends on the specific form of Hantavirus involved and clinical manifestations (5). In China, the Hantaan virus (HTNV) and the Seoul virus (SEOV) are the two main epidemic strains and are geographically quite stable; these induce severe and mild forms of HFRS, respectively (6–8). There are no specific antivirals for HFRS as yet. Vaccines with known efficacy to confer protective immunity against hantaviruses have reduced the prevalence of HFRS (9, 10); however, their long-term efficacy has yet to be confirmed, and vaccine coverage is far from satisfactory.

The specific immune status of pregnant women might increase their susceptibility to hantavirus infection, which presents specific clinical manifestations and influences the outcomes of both the mother and fetus (11). The epidemiological picture and clinical severity of hantavirus infections in pregnant women has yet to be fully described. This is because of the scarcity of case reports describing HFRS in pregnancy, a condition that poses significant difficulties for clinical management. In addition, inconsistent maternal and fetal outcomes have been reported previously (12–17). In previous case reports, results indicated that hantavirus infection in pregnant women resulted in serious effects on maternal and fetal outcomes, such as intrauterine fetal distress, death, abortion, or neonate death (18). Nevertheless, the majority of the existing data are based on case reports (Supplementary Table 1). A previous case report highlighted the fact that pregnant patients with HTNV infection present with a more severe clinical course, although the strength of this study was limited by a small sample size (11). The acquisition of additional clinical data, featuring detailed descriptions of hantavirus infections during pregnancy, is vital if we are to better understand the epidemiological and clinical characteristics of pregnant women and how it impacts maternal and fetal outcomes.

The aim of the current retrospective study, therefore, was to investigate the diagnosis, treatment, and prognostic prediction of the characteristics and outcomes of HFRS in pregnant patients. We compared these data with non-pregnant women with HFRS to highlight the specific clinical features of HFRS in pregnant women so that we can improve the early diagnosis of critical cases and initiate comprehensive therapy.

## Patients and Methods

### Study Population

This retrospective study was conducted in the First Affiliated Hospital of Xi'an Jiaotong University (a large tertiary-care hospital located in Xi'an, Shaanxi, Northwest China) with the approval of the institutional ethics review committee (Reference. XJTU1AF2021LSK-286). Patients diagnosed with HFRS were screened from inception to May 1^st^ 2021. Patients diagnosed with HFRS during pregnancy were enrolled, and a cohort of non- pregnant women of childbearing age (15–49 years old) with HFRS were also included as controls. We excluded patients if they had been diagnosed with other kidney diseases, cancer, cardiovascular disease, hematological disease, liver diseases, and if they used anticoagulants or antiplatelet agents.

### Diagnostic Criteria of HFRS

The diagnosis was defined according to criteria from the Prevention and Treatment Strategy of HFRS published by the Ministry of Health, PR China. The criteria includes clinical manifestations (e.g., fever, headache, low back pain, orbital pain, hemorrhage, hypotension, and acute renal dysfunction), epidemiological data (potential exposure history to secretions of wild rodents within 2 months before the onset of illness), and laboratory examinations. Laboratory criteria included serological positivity of IgM and/or IgG antibodies against hantavirus. The specific antibodies against Hantaan virus in serum during acute phase were detected by an indirect immunofluorescence test prior to 2010 or enzyme-linked immuno-sorbent assay from 2010.

### Data Collection

Demographic, clinical, and laboratory data were retrieved from the electronic medical records. Demographic data included age, gestational history, gestational week, HFRS vaccination history, and present address. Clinical data included onset season, days from the onset of fever to admission, chief complains, vital signs on admission, hospital stay, and severe HFRS-related complications including hemorrhage, secondary infection, hepatic injury, kidney rupture, pulmonary edema, sepsis, multiple organ dysfunction syndrome (MODS), disseminated intravascular coagulation (DIC), and acute respiratory distress syndrome (ARDS).

Laboratory parameters included routine blood and urinary analyses, kidney function and liver function; these tests were carried out routinely by auto-analyzers. Fetal heart rates were monitored routinely during hospitalization and echocardiography was applied to monitor morphological and functional development.

Management and clinical outcomes were also collected. Follow-up after discharge was carried out by telephone survey and data retrieval from the electronic medical system. Pregnancy outcomes were defined according to the European Medicines Agency (EMA) and Centers for Disease Control and Prevention (CDC) guidelines. The major outcome concerned with fetuses/neonates was death or fetal distress during the clinical course of HFRS and birth defects prior to birth, at birth, or at any time after birth.

### Clinical Classification and HFRS-Related Complications

Disease severity was classified into mild, moderate, severe, and gravis types according to the diagnosis criteria for the prevention and treatment strategy of HFRS published by the Ministry of Health, China: (1) mild, max temperature (Tmax) <39°C, mild toxemic symptoms, kidney injury without oliguria and hypotension; (2) moderate, Tmax in the range of 39–40°C, obvious effusion (bulbar conjunctiva), hemorrhage (skin and mucous membranes), hypotension, and significant kidney injury with uremia and typical oliguria (urinary output of 50–500 mL/day); (3) severe, Tmax ≥40°C, severe effusion (bulbar conjunctiva and either peritoneum or pleura effusion), hemorrhage (skin and mucous membranes ecchymosis or cavity hemorrhage), hypotension, kidney injury with severe uremia and oliguria lasting for ≤ 5 days or anuresis lasting for ≤ 2 days; (4) gravis, severe refractory shock (≥2 days), visceral hemorrhage, severe kidney damage (oliguria >5 days, or anuria >2 days, or urea nitrogen >42.84 mmol/L, or creatinine >353.6 umol/L, or creatinine > three times the baseline) or other serious complications such as heart failure, pulmonary edema, respiratory failure, severe secondary infection, cerebral edema or cerebral hemorrhage or even MODS. Definitions for the related complications were in accordance with published parameters (6).

### Statistical Analysis

Statistical analysis was carried out using SPSS 22.0 version software (SPSS Inc., Chicago, IL, USA). Continuous variables are expressed as means (±standard deviation) or median (range); normality was analyzed by the Kolmogorov-Smirnov's test. Continuous variables were compared using the *t* test or Mann-Whitney *U* test. Categorical variables are expressed as frequencies and percentages, and the Chi-squared test or Fisher's exact test was applied to compare the two groups of patients. Logistic regression analysis was applied to assess the clinical correlations. *P*-values <0.05 were considered as statistically significant.

## Results

### Demographic Characteristics of Patients With HFRS

**Figure 1:**
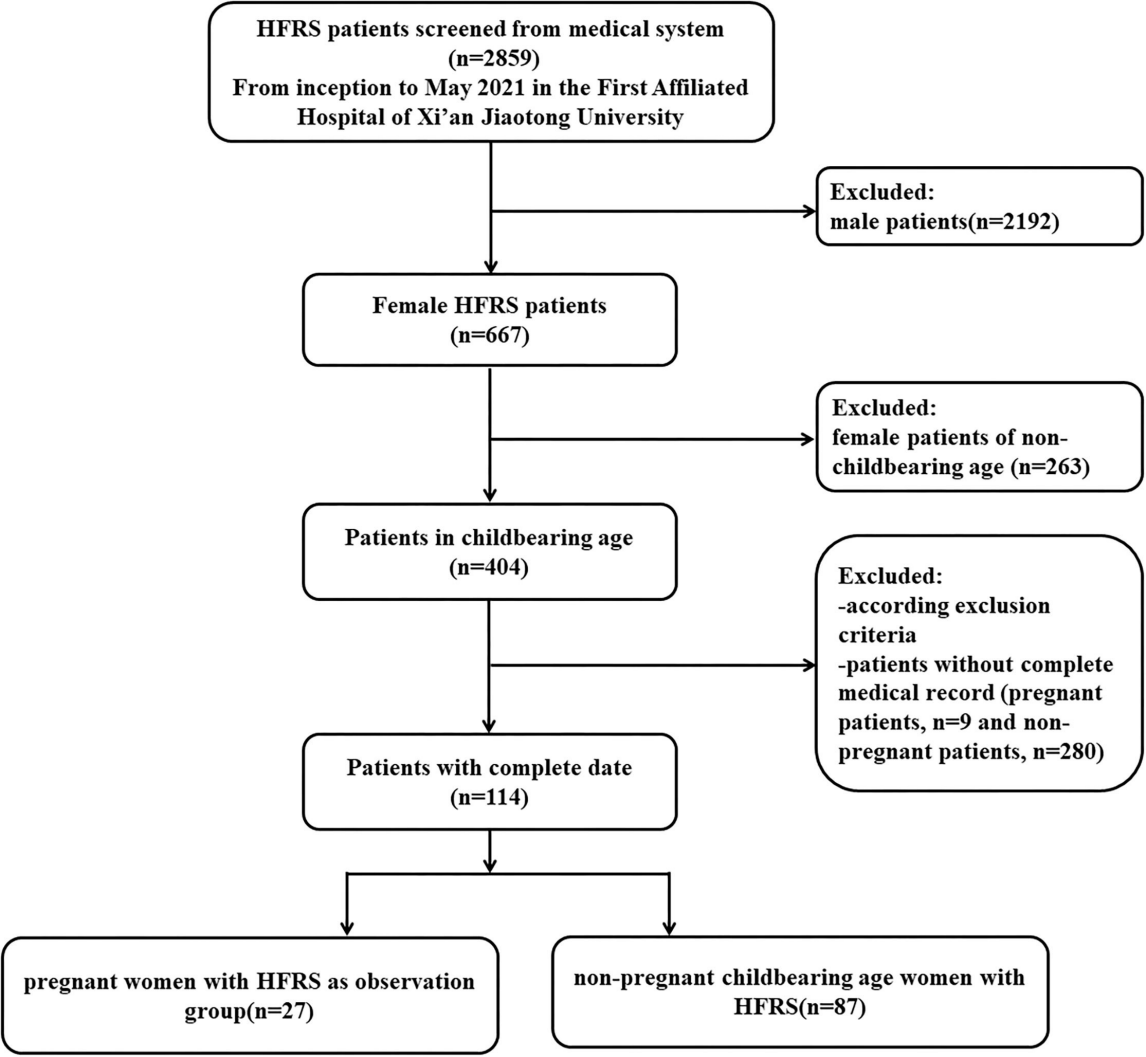
Flowchart of patient selection.

As shown in the flowchart of patient selection (Figure 1), among a total of 2,859 HFRS patients screened from inception to May 2021 in the First Affiliated Hospital of Xi'an Jiaotong University, 667 patients were female (the ratio of male to female cases was approximately 3.29:1). According to the inclusion and exclusion criteria, we enrolled 27 pregnant women (mean age 27.56 ± 4.81 years) diagnosed with HFRS, who had a complete medical record, laboratory tests, and outcome information. We also included 87 non-pregnant female patients as controls (mean age 33.32 ± 9.75 years).

Among the enrolled patients, 25 out of 27 pregnant patients and 78 out of 87 non-pregnant patients, were from Shaanxi, an endemic area for HTNV infection. In addition, most of the patients were reported an onset ranging from November to January of the following year (62.96% in the pregnant group; 68.97% in the non-pregnant group), the epidemic season of HFRS in Shaanxi. Of the 27 pregnant patients, seven were in the first trimester, 8 in the second trimester, and 12 in the third trimester. All 27 cases were previously healthy without any recorded complications. Only one pregnant patient and four non-pregnant patients accepted more than three doses of the HFRS vaccine prior to pregnancy.

### Clinical Characteristics of the Pregnant Women With HFRS

Detailed clinical characteristics of the 27 pregnant patients are presented in Table 1. Maternal ages ranged from 17 to 39 years and the gestational ages at the time of diagnosis ranged from 6 to 39 weeks. Among the 27 pregnant patients, 15 cases were classified as critical types, including seven (25.9%) severe cases and eight (29.6%) gravis cases; the remaining cases included seven (25.9%) mild cases and five (26.3%) moderate cases. Furthermore, the proportions of critical cases in the first, mid, and late trimester of pregnancy was 13.3% (2/15), 33.3% (5/15), and 53.4% (8/15), respectively. With further analysis, we found that patients had a higher risk of severity as the pregnancy progressed (*P* = 0.000, X_2_ = 37.880, Table 2).

Table 1General information of the 27 pregnant women with HFRS.

**Table:** 

**Patient No**.	**Age**	**Gestational**	**History**	**Onset season**	**Time from onset**	**Clinical phase of**	**Findings in febrile phase**			
	**(Years)**	**period (weeks)**	**of gestation**		**to admission**	**HFRS at admission**				
					**(days)**		**Max emperature**,	**Headache**	**Low back**	**Orbital**
							**duration (days)**		**pain**	**pain**
1	39	16	G8P6	1962/12	8	Overlap of febrile and oliguric phase	38.0°C, 10	Yes	No	No
2	38	12	G4P3	1964/12	6	Oliguric phase	39.8°C, 7	Yes	Yes	Yes
3	24	28	G4P3	1971/11	7	Oliguric phase	38.0°C, 10	Yes	No	No
4	29	16	G1P0	1981/11	4	Febrile phase	38.6°C, 8	Yes	Yes	Yes
5	25	12	G1P0	1983/10	3	Oliguric phase	39.0°C, 8	Yes	Yes	Yes
6	28	29	G1P0	1983/12	3	Febrile phase	40.3°C, 7	Yes	Yes	Yes
7	26	29	G1P0	1984/12	5	Febrile phase	40.5°C, 6	Yes	Yes	Yes
8	28	26	G2P1	1990/11	7	Oliguric phase	40.5°C, 7	Yes	Yes	Yes
9	23	26	G1P0	2004/12	10	Polyuric phase	38.5°C, 5	Yes	Yes	Yes
10	28	32	G2P1	2004/12	5	Febrile phase	38.5°C, 5	Yes	Yes	No
11	25	25	G1P0	2005/11	5	Oliguric phase	38.0°C, 4	Yes	Yes	No
12	35	32	G2P1	2006/05	3	Oliguric phase	38.5°C, 2	Yes	Yes	No
13	28	30	G3P1	2007/10	1	Hypotensive phase	40.0°C, 6	Yes	Yes	No
14	32	6	G2P1	2008/12	4	Febrile phase	39.0°C, 7	Yes	No	No
15	27	35	G3P2	2010/12	4	Overlap of febrile and hypotensive phase	39.5°C, 6	Yes	Yes	No
16	17	39	G1P0	2012/6	4	Febrile phase	39.5°C, 5	No	No	No
17	23	36	G2P1	2014/12	6	Febrile phase	39.8°C, 8	Yes	Yes	Yes
18	25	8	G1P0	2015/04	5	Overlap of febrile and oliguric phase	39.2°C, 5	Yes	No	No
19	23	30	G1P0	2015/08	7	Oliguric phase	39.0°C, 5	Yes	Yes	No
20	23	27	G2P1	2015/12	5	Oliguric phase	38.6°C, 5	Yes	Yes	No
21	24	17	G2P1	2016/02	3	Febrile phase	39.6°C, 5	Yes	Yes	Yes
22	28	27	G2P0	2016/10	5	Febrile phase	39.2°C, 7	No	Yes	No
23	28	27	G2P0	2017/12	4	Febrile phase	40.5°C, 6	Yes	Yes	Yes
24	27	32	G2P1	2019/01	6	Polyuric phase	39.8°C, 4	Yes	Yes	No
25	31	22	G2P0	2020/9	5	Febrile phase	39.7°C, 5	Yes	Yes	No
26	28	32	G3P1	2020/11	5	Febrile phase	40.2°C, 8	Yes	Yes	No
27	33	30	G2P0	2020/11	4	Febrile phase	38.5°C, 9	Yes	No	No

**Table:** 

**Patient No**.	**Findings in febrile phase**			**Hypotensive shock^a^ (BP/ mmHg)**	**Oliguria, min urine volume (mL/24 h)**	**Polyuria, max urine volume (mL/24 h)**	**Multiple stages overlapping**
	**Face-neck-chest congestion**	**Hemorrhagic tendency**	**Chemosis, grade**				
1	Yes	Yes	2 (moderate)	No	Yes, 20	Yes ,3,500	Overlap of febrile and oliguric phase
2	Yes	Yes	2 (moderate)	No	Yes, 400	Yes ,4,100	No
3	No	No	No	No	No, 1800	Yes, 2400	No
4	Yes	Yes	2 (moderate)	No	No, 600	Yes, 2,900	No
5	Yes	Yes	1 (mild)	No	No, 1450	Yes, 5,330	No
6	Yes	Yes	2 (moderate)	No	Yes, 430	Yes, 3,600	Overlap of febrile and oliguric phase
7	Yes	Yes	3 (severe)	Yes (80/60)	Yes, <200	Yes, 4,600	Overlap of febrile and hypotensive phase
8	Yes	Yes	2 (moderate)	Yes (80/50)	Yes, 20	Yes, 4,090	Overlap of febrile and hypotensive phase
9	No	Yes	1 (mild)	Yes (80/60)	Yes, 500	Yes, 4,500	Hypotensive and oliguric phases
10	Yes	Yes	2 (moderate)	No	Yes, 400	Yes, 6,340	No
11	Yes	Yes	2 (moderate)	Yes (80/44)	Yes, 200	Yes, 4,600	Overlap of febrile and hypotensive phase
12	Yes	Yes	2 (moderate)	No	Yes, 100	Yes, 5,200	No
13	No	Yes	2 (moderate)	Yes (80/50)	Anuria	Anuria	Overlap of febrile, hypotensive and oliguric phases
14	No	Yes	1 (mild)	No	No, 2310	Yes, 4,050	No
15	No	Yes	3 (severe)	Yes (70/40)	Yes, Anuria	Yes, 4,000	Overlap of febrile and hypotensive phase
16	Yes	No	1 (mild)	No	No, 1040	Yes, 5,300	No
17	Yes	No	1 (mild)	No	No, 1200	Yes, 5,100	No
18	Yes	Yes	1 (mild)	No	Yes, 300	Yes, 3,370	Overlap of febrile and oliguric phase
19	No	Yes	1 (mild)	No	Yes, <50	Yes, 4,850	No
20	Yes	Yes	No	No	No, 760	Yes, 5,400	No
21	Yes	No	2 (moderate)	No	No, 1520	Yes, 5,520	No
22	Yes	No	3(severe)	No	Yes, 175	Yes, 5,400	No
23	Yes	Yes	2(moderate)	Yes (80/50)	No, 1100	Yes, 7,570	Overlap of febrile and hypotensive phase
24	No	Yes	2 (moderate)	Yes (70/40)	No, 1630	Yes, 6,410	No
25	Yes	Yes	1(mild)	No	No, 1400	Yes, 4,990	No
26	Yes	Yes	2 (moderate)	No	No, 960	Yes, 5,930	No
27	No	Yes	1 (mild)	No	No, 690	Yes, 4,945	No

**Table:** 

**Patient No**.	**Severe complications**	**Hemodialysis**, **mode**	**Clinical classification of HFRS^b^**	**Duration of hospitalization**	**Hantavirus diagnostics**		**Max WBC count (×10** ^**9**^ **/L)**	**Max LYM count (×10** ^**9**^ **/L)**	**Max NEUT count (×10** ^**9**^ **/L)**
					**Anti-HFRS IgM**	**Anti-HFRS IgG**			
1	secondary infection; pulmonary edema; hepatic injury; arrhythmia	No	Severe	12 days	Positive	Positive	31.5	13.02	38.4
2	secondary infection; pulmonary edema	No	Severe	15 days	Positive	Positive	30.4	6.69	20.98
3	No	No	Mild	12 days	Positive	Positive	7.4	1.78	5.62
4	No	No	Mild	6 days	Positive	Positive	14.3	2.86	10.44
5	No	No	Mild	11 days	Positive	Positive	10.4	3.12	7.07
6	secondary infection; pulmonary edema; arrhythmia	No	Gravis	16 days	Positive	Positive	26.8	6.43	20.37
7	secondary infection; pulmonary edema; arrhythmia	No	Gravis	15 days	Positive	Positive	21	2.1	16.59
8	secondary infection; pulmonary edema; hepatic injury	No	Gravis	21 days	Positive	Positive	10.6	2.8	8.16
9	No	No	Severe	6 days	Positive	Positive	10.2	2.47	4.13
10	No	No	Moderate	15 days	Positive	Negative	11.7	-	-
11	hepatic injury	No	Severe	12 days	Positive	Negative	29.1	7.45	14.07
12	hepatic injury; MODS	No	Gravis	8 days	Positive	Negative	16.8	3.09	12.2
13	hemorrhage; secondary infection; MODS; DIC	Yes, CRRT	Gravis	1 days	Positive	Negative	82.23	22.8	46.5
14	No	No	Mild	11 days	Positive	Positive	5.6	2.08	4.05
15	hemorrhage; pulmonary edema	Yes, CRRT	Gravis	11 days	Positive	Positive	26.75	13.62	13.76
16	No	No	Mild	11 days	Positive	Positive	11.41	1.97	9.51
17	secondary infection; sepsis; MODS	No	Gravis	10 days	Positive	Negative	13.51	4.99	17.25
18	No	No	Moderate	10 days	Positive	Positive	14.16	5.26	7.4
19	secondary infection; MODS; pulmonary edema; ARDS	Yes, CRRT	Gravis	11days	Positive	Positive	16.05	4.08	10.81
20	No	No	Mild	5 days	Positive	Positive	10.73	5.04	5.04
21	No	No	Moderate	13 days	Positive	Negative	20.08	3.8	11.85
22	No	No	Severe	20 days	Positive	Negative	23.65	7.04	18.64
23	hepatic injury	No	Severe	14 days	Positive	Negative	13.96	4.14	9.61
24	secondary infection	No	Moderate	9 days	Positive	Negative	9.53	3.21	6.15
25	No	No	Mild	6 days	Positive	Positive	9.06	2.92	5.38
26	sepsis	No	Severe	8 days	Positive	Negative	18.67	6.01	15.6
27	secondary infection; pulmonary edema	No	Moderate	12 days	Positive	Negative	36.57	4.44	31.78

**Table:** 

**Patient No**.	**Max hemoglobin (g/L)**	**Min hemoglobin (g/L)**	**Min PLT count (×10^9^/L)**	**PDW(fL)**	**Proteinuria^c^**	**Min serum albumin (g/L)**	**Max serum nitrogen (mmol/L)**	**Max serum creatinine (μmol/L)**	**Max serum ALT (U/L)**	**Max serum AST (U/L)**
1	80	53	120	-	+	29.8	10.53	254	96	100
2	104	77	120	-	+++	26.8	11.64	301	25	36
3	132	128	107	-	+	-	7.65	169	-	-
4	88	85	80	-	+++	29.8	11	243	34	37
5	95	90	60	-	+	30.3	18.3	178	42	63
6	82.9	78.9	30	-	>+++	-	28.05	289.10	-	-
7	83	80	46	-	+++	23.6	12.33	489.10	17	17
8	72	70	40	-	>+++	21.6	13.5	813.28	100	98
9	95	87	105	18.1	Negative	21.6	33.65	415.8	10	46
10	116	100	92	-	++	-	13.6	193	-	-
11	102	78	17	20.3	+++	23.8	31.74	244	13	96
12	138	89	14	14.5	+++	30.9	18.30	310	200	402
13	150	93	17	17.3	+++	30	11.60	248	2908	4600
14	108	85	36	17.0	++	34.3	6.03	121.6	60	81
15	168	75	18	22	+++	17.5	18.21	291.86	60	141
16	110	81	38	11.2	+++	25.3	11.4	255	149	46
17	152	75	36	23.9	+	24.1	14.75	164	22.0	45.9
18	140	113	39	16.3	++	29.7	7.14	121	50.8	143.5
19	158	88	50	14.4	+++	22.5	16.57	655	27	47
20	115	77	30	17.8	+++	23.3	7.81	151	19	52
21	140	105	86	15.0	+++	28.6	17.16	377	39.2	92.7
22	144	94	45	16.8	++	25.9	17.54	460	36	82
23	119	81	65	16.5	+	20.2	14.19	461	263	287
24	90	86	45	17.5	++	35.5	6.18	93	22	36
25	91	81	82	16.3	+	21.3	7.65	169	42	35
26	148	83	34	17.7	+++	19.6	8.03	164	104	182
27	114	84	14	16	+++	20.7	16.28	494	47	49

*BP, blood pressure; WBC, white blood cell; LYM, lymphocyte; NEUT, neutrophile granulocyte; PLT, platelet; PDW, platelet distribution width; ALT, alanine transaminase; AST, aspartate aminotransferase; CRRT, continuous renal replacement; HFRS. Hemorrhagic fever with renal syndrome, MODS, multiple organ dysfunction syndrome; ARDS, acute respiratory distress syndrome; DIC, dissinted intrvsculr cogultion*.

a *Defined as the presence of blood pressure <90/60 mmHg in Hypotension shock phase*.

b *Classification of the clinical severity of HFRS were accorded to published paramenters*.

c *On admission. Reference values, ALT (9–40) U/L; AST (15–40) U/L; Hemoglobin (110–150) g/L (female); Platelets (100–300) ×10^9^/L; Serum albumin (35–55) g/L; Serum creatinine (41–73) μmol/L (female); Urea nitrogen (2.6–7.5) mmol/L (female); WBC count (4.0–10) ×10^9^/L; LYM count (1.1–3.2) ×10^9^/L; NEUT count (1.8–6.3) ×10^9^/L*.

**Table 2:** Relationship between duration of pregnancy and severity of HFRS.

	**First trimester of pregnancy**	**Mid trimester of pregnancy**	**Late trimester of pregnancy**	***X^**2**^***	***P*-value**
**Clinical classification of HFRS (** ***n*** **/%)**
Mild type	3 (42.8)	3 (37.5)	1 (8.3)	37.880	0.000
Moderate type	2 (28.6)	0 (0.00)	3 (25.0)		
Severe type	2 (28.6)	4 (50.0)	1 (8.3)		
Gravis type	0 (0.00)	1 (12.5)	7 (58.4)		

*HFRS, Hemorrhagic fever with renal syndrome*.

The majority of these patients presented typical clinical manifestations in the acute phase, such as headache, low back pain, face-neck-chest congestion, chemosis, skin or mucous hemorrhage; these manifestations were similar with those of non-pregnant women of childbearing age with HFRS. Thrombocytopenia was observed in 23 pregnant patients; 95.8% (23/24) of pregnant patients experienced hypoalbuminemia and 33.3% (9/27) experienced anemia during the process of disease, respectively. Liver injury was observed in 13 patients. Hyperleukocytosis with a leukocyte count higher than 10 × 10^9^/L occurred in 85.2% (23/27) of pregnant women. Eight patients (29.6%) experienced hypotensive shock; the lowest systolic and diastolic blood pressures were 79 ± 6 mmHg and 49 ± 8 mmHg, respectively. In addition, proteinuria was detected in 26 patients (96.3%); 11 cases (40.7%) received transfusions of albumin and plasma for treatment. Varying degrees of renal dysfunction were evident in these pregnant patients. The maximal level of serum nitrogen ranged from 6.03 to 33.65 mmol/L, and the maximal level of serum creatinine ranged from 93 to 813.28 mmol/L. Furthermore, continuous renal replacement therapy (CRRT) was applied in three patients to alleviate pulmonary edema or symptoms of hyper-azotemia.

With regards to HFRS-related complications, the incidences of MODS, secondary infection (pulmonary or urinary infection), pulmonary edema, DIC and ARDS, were 14.8% (4/27), 33.3% (9/27), 29.6% (8/27), 3.7% (1/27) and 3.7% (1/27), respectively.

### Comparisons Between Pregnant Patients and Non-pregnant Patients With HFRS

To further investigate the specific clinical characteristics of pregnant patients, we performed a direct comparison with non-pregnant women of childbearing age with HFRS. There were no significant differences in terms of the history of vaccination, maximal temperature, or blood pressure on admission in the two groups (Table 3). Compared to non-pregnant female patients, pregnant patients with HFRS presented with a more critical clinical course (55.5 vs. 21.8%, *P* = 0.008). Furthermore, pregnant patients also experienced far more severe complications (55.6 vs. 46.0%, *P* = 0.384), especially pulmonary edema, and significant hyperleukocytosis (85.2 vs. 70.1%, *P* = 0.120). However, there were no significant differences between the two groups with regards to lymphocyte counts, platelet counts, platelet distribution width, urea nitrogen and serum creatinine (*P* > 0.05). However, pregnant females tended to have much more frequent anemia (33.3 vs. 4.6%, *P* <0.001) and hypoalbuminemia (83.33 vs. 60.92%, *P* = 0.040) compared with the non-pregnant group.

**Table 3:** Comparison between pregnant women and non-pregnant female patients with HFRS.

	**Pregnant**	**Non-pregnant**	***P*-value**
	**patients**	**female patients**	
**Present address**			
City	6	25	0.506
Country	21	62	
**Patients with source**			
Shaanxi or Xi'an	25	78	0.937
Other places outside Shaanxi	2	9	
**HFRS vaccination history**			
Yes	1	4	1.000
No	26	83	
Time from onset to admission (day)	4.96 ± 1.65	4.98 ± 2.08	0.158
Average stay	11.15 ± 4.41	10.98 ± 4.81	0.516
**ICU admission**			
Yes	1	3	1.000
No	26	84	
Tmax	39.27 ± 0.80	39.25 ± 0.78	0.429
Hypotensive shock (*n*/%)	8 (29.6)	17 (19.5)	0.268
Severe complications (*n*/%)	15 (55.6)	40 (46.0)	0.384
Hemorrhage	2 (7.4)	6 (6.9)	1.000
Secondary infection	9 (33.3)	32 (46.8)	0.744
Hepatic injury	7 (25.9)	20 (23.0)	0.754
Sepsis	1 (3.7)	2 (2.3)	0.559
MODS	4 (14.8)	4 (4.6)	0.166
Arrhythmia	3 (11.1)	3 (3.4)	0.144
Pulmonary edema	8 (29.6)	5 (5.7)	0.002
ARDS	1 (3.7)	2 (2.3)	0.559
DIC	1 (3.7)	2 (2.3)	0.559
Encephalopathy	0 (0)	1 (1.1)	1.000
Multiple stages overlapping (*n*/%)	10 (35.7)	19 (21.8)	0.113
Hyperleukocytosis (*n*/%)	23 (85.2)	61 (70.1)	0.120
^#^Maximum leukocyte counts, ×10^9^/L	10.67–25.20	9.10–18.19	0.145
^#^Nadir platelet count, ×10^9^/L	31–76	24–79	0.122
^#^Platelet distribution width, fL	16.08–17.78	16.0–18.2	0.929
Thrombocytopenia (*n*/%)	22 (81.5)	70 (80.5)	0.906
Anemia at admission (*n*/%)	9 (33.3)	4 (4.6)	0.000
^#^Nadir hemoglobin, g/L	79.5–89.5	92–109	0.238
Hypoalbuminemia at admission (*n*/%)	^*^23 (95.83)	73 (83.9)	0.24
Hypoalbuminemia during hospitalization *(n*/%)	^*^20 (83.33)	53 (60.92)	0.040
^#^Nadir serum albumin, g/L	21.60–29.73	26.90–30.20	0.034
Renal disfunction (*n*/%)	26 (96.3)	76 (87.4)	0.335
^#^Maximum urea nitrogen, mmol/L	10.53–17.54	7.48–23.00	0.066
^#^Maximum serum creatinine, umol/L	169–415.8	181.9–507.75	0.135
CRRT treatment (*n*/%)	3 (11.1)	4 (4.6)	0.440
^#^Maximum procalcitonin, ug/L	1.25–7.15	0.33–4.36	0.195
^#^Maximum C-reactive protein, mg/L	27.99–75.25	18.55–52.40	0.521
Clinical classification of HFRS (*n*/%)			
Mild type	7 (25.9)	50 (57.5)	0.008
Moderate type	5 (18.5)	18 (20.7)	
Severe type	7 (25.9)	7 (8.0)	
Gravis type	8 (29.6)	12 (13.8)	

*HFRS, Hemorrhagic fever with renal syndrome; ICU, intensive care unit; T, temperature; MODS, multiple organ dysfunction syndrome; ARDS, acute respiratory distress syndrome; DIC, dissinted intrvsculr cogultion; CRRT, continuous renal replacement. Date is expressed as number (%) and*

# *interquartile range*.

* *24 patients had albumin level during hospitalization*.

Since a significantly higher incidence of critical cases were observed in pregnant patients, we next investigated the factors associated with critical cases. To further analyze the risk factors associated with critical status, the patients were classified into two groups, patients with mild and moderate types were designated as a mild group, while patients with severe and gravis types were designated as a critical group. Next, we performed univariable logistic regression analysis using a range of variables, including age, gestational week, Tmax, duration of the febrile phase, systolic and diastolic blood pressure, the incidence of overlapping phase at admission, leukocyte counts, neutrophile granulocyte counts, platelet counts, hemoglobin level, albumin level, and urinary protein (Table 4). Maximum leukocyte count during the febrile phase was identified as a significant risk factor for disease severity [odds ratio (OR) =1.139, 95% confidence interval (CI) 1.004, 1.291, *P* = 0.043]; however, none of the variables analyzed generated a protective effect.

**Table 4:** Risk factors associated with pregnant female patients with critical HFRS.

**Variables**	**Univariable logistic regression**	
	**OR (95% CI)**	***p-*value**
Age	1.095 (0.919, 1.305)	0.311
Mid trimester of pregnancy	0.200 (0.026, 1.526)	0.121
Late trimester of pregnancy	0.833 (0.129, 5.396)	0.848
Late trimester of pregnancy vs. First and Mid trimester of pregnancy	2.286 (0.475, 11.003)	0.303
Tmax at febrile phase	2.327 (0.801, 6.760)	0.120
Duration of the febrile phase	1.018 (0.689, 1.504)	0.930
SBP	0.959 (0.889, 1.034)	0.277
DBP	0.949 (0.877, 1.026)	0.185
Incidence of overlapping phase at admission	9.625 (0.980, 94.540)	0.052
WBCmax at febrile phase	1.139 (1.004, 1.291)	0.043
WBC ≥ 15 × 10^9^/L	22.000 (2.18, 221.974)	0.009
Nmax at febrile phase	1.147 (0.984, 1.338)	0.079
PLTmin at febrile phase	0.996 (0.971, 1.021)	0.733
HGB level at admission	1.012 (0.983, 1.041)	0.437
ALB level at admission	0.830 (0.679, 1.016)	0.071
Urine protein ≥ 3+ at admission	2.800 (0.582, 13.478)	0.199

*HFRS, Hemorrhagic fever with renal syndrome; T, temperature; SBP, systolic blood pressure; DPB, diastolic blood pressure; WBC, white blood cell; N, neutrophile; PLT, platelet; HGB, hemoglobin; ALB, albumin*.

### Maternal and Fetal Outcomes

Twenty-six of the 27 pregnant patients (96.3%) progressed to the poly-uric phase with a maximal urinary volume of 2400–7570 mL/24 h and recovered completely after reasonable and timely comprehensive treatment. Only one pregnant patient deteriorated rapidly and experienced fetal death when diagnosed as a gravis type and died of DIC on the second day of hospitalization. None of the pregnant patients showed evidence of abnormal fetal development, fetal distress, or pregnancy complications before admission. It was not possible to acquire outcomes for five fetuses owing to their loss during follow-up. Fifteen live births were reported, including one premature delivery owing to intrauterine distress (patient number 17), there were two other cases of intrauterine fetal death (patient numbers 11 and 13). In addition, one malformation was reported by four-dimensional echocardiography and termination was conducted after recovery (patient number 22). Unfortunately, selective termination was chosen by three patients after discharge due to concerns relating to the “potential effect” of HFRS and medications administered during treatment.

### The Influence of HFRS on the Fetus

It is noteworthy that all adverse fetal events occurred in critical cases during the mid and late trimesters of pregnancy (*P* = 0.047, X_2_ = 4.909, Table 5). In term of vertical transmission, clinical manifestations associated with HFRS were not reported in the newborns, although no additional tests for HTNV were performed on the infants at the time of delivery or after discharge.

**Table 5:** Relationship between fetal adverse events and severity of HFRS.

	**Adverse**	**No adverse**	***X^**2**^***	***P*-value**
	**events**	**events**		
	**occur**	**occurred**		
**Fetal adverse events (** ***n*** **/%)**
Mild and moderate type	0	12	4.909	0.047
Critical (severe and gravis type)	5	10		

*HFRS, Hemorrhagic fever with renal syndrome*.

## Discussion

Pregnant women can be infected with hantaviruses *via* contact with secretions from infected rodents or food contaminated by animal excreta. Cases of pregnant women infected with hantaviruses, although scarce, have been reported across endemic regions. Regarding the potential threat to both the mother and fetus, concerns have been raised among infectious disease doctors and gynecologists when a pregnant woman is complicated with HFRS. This study, to our knowledge, reports the largest number of pregnant women with HFRS in the English literature and analyzed the influence of this disease on pregnant women and their fetuses. Our findings enrich the understanding of maternal and fetal outcomes associated with hantaviruses infection, and the factors associated with disease severity. We found that pregnant patients with HFRS present with more severe clinical types, especially those with hyperleukocytosis. Furthermore, the incidences of hypoalbuminemia and anemia were more common in pregnant patients, when compared with non-pregnant patients.

Pregnant women might be infected by the inhalation of aerosols or dust particles from hantavirus-contaminated rodent secretions. The majority of previous case reports relating to pregnant women with HFRS are from endemic regions of HTNV and SEOV, such as China and South Korea; these areas are associated with a high incidence of HFRS (19). This study, consistent with the main findings of previous reports, showed a high likelihood of severity in pregnant women, especially those in the third trimester (11). In current study, 55.4% of pregnant patients were critical compared to only 21.8% in non-pregnant patients; this was consistent with the presumed endemic hantavirus species in Shaanxi. In addition, the diagnosis was determined by serological positivity of IgM and/or IgG antibodies against hantavirus; we did not detect or isolate the virus. However, epidemiological studies have confirmed that HTNV infection is the major causative strain of HFRS in Shaanxi Province, one of the most severely affected regions in China, HFRS patients in this district present more severe manifestations (20, 21). In addition, the normal physiological changes during pregnancy (i.e., an expansion in intravascular volume) would also affect the progression of HFRS. To meet the metabolic demands of the mother and her fetus, the blood volume increases by 40–50% from 6 to 34 gestational weeks. This hemodynamic change leads to the increment of cardiac output and glomerular filtration rate. When pregnant women were infected by HTNV, plasma extravasation would cause more serious hypovolemia and systemic damage, especially kidney injury. Taking these factors together, patients in the third trimester of pregnancy are associated with a high risk of a severe status and should be taken seriously.

Pregnant women experience significant anatomical and physiological changes (22). Hypoalbuminemia, arising from increasing nutrient demand, expanded intravascular volume, and decreasing protein synthesis, is associated with outcomes of pregnant women and the developing fetus (23). Negative relationships have been reported between serum albumin level and pregnancy complications, such as preterm labor, fetal growth retardation, and placenta abruption (24). Previous investigations have found that hypoalbuminemia is common in HFRS (4, 9); some cohorts have exhibited a close correlation between serum albumin level and both disease severity and mortality (25, 26). There are several factors attributed to hypoalbuminemia. Firstly, vascular leakage of serum protein due to increased vascular permeability and positive urinary protein, can cause the loss of albumin. Secondly, the reduced oral intake and increased catabolism of protein would also influence the level of serum albumin. In addition to hypoalbuminemia, a significantly higher frequency of anemia was detected in pregnant patients (33.3 vs. 4.6%, *p* <0.001). In a previous case report, Ji reported a clearly lower level of hemoglobin and serum albumin, although the sample size limited the clinical significance. Anemia during pregnancy is also a significant health problem; this has been confirmed to be associated with intrauterine growth retardation, premature birth, and elevated maternal and prenatal mortality (27). However, the frequency of anemia was not higher than that reported in the general pregnant population, ranging from 5.4% in the United States to more than 80% in developing countries (28). Despite further univariable logistic regression analysis, the association of anemia with the severity of disease was not confirmed. Thus, the anemia observed in current cohort may be attributed to gestation. Compared with non-pregnant patients, pregnant women with HFRS were also associated with a high risk of hypoalbuminemia and anemia; those conditions would have a potentially reversible impact on pregnancy outcomes. Furthermore, when considering HFRS-related complications, the incidence of pulmonary edema was significantly higher than those of non-pregnant patients; this was also attributed to hypoalbuminemia and associated with disease severity.

As an acute viral disease, HFRS is widely believed to present a systematic inflammatory response (29). The current study showed that compared with patients with the mild and moderate types of HFRS, pregnant women with the critical type suffered far more from marked hyperleukocytosis. In a previous investigation, the association of leukocyte counts with a severe clinical type was not investigated in pregnant patients; this was due to the scarcity of cases. In the general population, leukocyte count increases gradually with the exacerbation of disease severity (26). However, the pathogenesis of hyperleukocytosis has yet to be fully elucidated. The overproduction of inflammatory cytokines has been commonly reported in patients with HFRS and has given rise to the hypothesis that a “cytokine storm” may play a pivotal role in the pathogenesis of this disease. At present, there is no specific therapy for HFRS. Therefore, hyperleukocytosis, especially in patients with leukocyte counts higher than 15 × 10^9^/L may indicate a potential tend with disease severity and facilitate the earlier identification of patients at risk of developing the severe and gravis types of HFRS. Previous studies have not identified risk factors for a critical status in pregnant women. Such results would help physicians to understand and monitor pregnant patients with HFRS.

The incidence of adverse fetal events was more likely related to the severe status of pregnant women with HFRS. Previous literature showed that the cases with fetal adverse events were all classified into severe or critical type (13, 15, 16). In current cohort, three cases resulted in intrauterine fetal death. In a recent review article, Lu et al. (18) reported a mortality rate of 31.8% with HTNV/SEOV; this is much higher than previous studies reported from Chinese researchers involving large sample size. Duan et al. (30) reported three deaths in 48 cases, while Lu et al. (31) reported four deaths in 18 cases; in present study we recorded three deaths in 27 cases. In a previous review, the rate was calculated by collectively pooling data from single case reports and case series; this practice would have overestimated the mortality rate considering the tendency to report severe cases. The mortality rate determined in current investigation remained within the ranges reported for the general population (1); this rate is mainly attributed to the early diagnosis and awareness of the risk of HFRS during pregnancy. Moreover, majority of the pregnant women enrolled were diagnosed after 2000; since then, CRRT has been extensively applied in treatment of HFRS; three patients from the present cohort underwent CRRT treatment. In addition, the close association of fetal adverse events with gestation weeks needs to be investigated further in pregnant women, especially in later trimester. Hence, early diagnosis and appropriate treatment are very important for the prognosis of HFRS.

Although hypotension shock, acute kidney injury, or even visceral hemorrhage occurred, induced delivery has also been reported; most pregnant women with HFRS could continue their pregnancy uneventfully and recover without sequelae with appropriate treatment (11, 18, 32). In line with the general HFRS population, 26 pregnant women, including 14 critical cases, were cured successfully in this cohort. More than 50% were diagnosed being in the febrile or hypotensive phase and 40.7% received rapid fluid replacement and transfusion of blood product. The compensation of hypovolemia and the improvement of organ perfusion are pivotal for the successful rescue of HFRS. For patients with life-threatening complications, such as pulmonary edema and hypervolemia, the application of CRRT can save lives by relieving hypervolemia or pulmonary edema. More importantly, close monitoring of fetal life signs and the appreciation of obstetric disposition are pivotal to both the mother and fetus during pregnancy. By reviewing the previous literature, we found that four pregnant women with HFRS were complicated with severe fetal adverse events but still recovered fully after terminating the pregnancy (13, 15, 16, 33). Of the three pregnant women experiencing intrauterine fetal death in current study, the two patients who selected timely termination recovered completely; However, the remaining patient refused to terminate pregnancy and subsequently died of DIC. Previous reports described five pregnant women who experienced hypervolemia and pulmonary edema but still gave birth to healthy infant (11, 14). Consistent with a previous study related to SEOV infection, a striking but transient liver injury was evident in this cohort (34). Two pregnancy-related diseases affecting mainly the liver, acute fatty liver of pregnancy (AFLP) syndrome and haemolysis, elevated liver enzymes and low platelet count (HELLP) syndrome, need urgent delivery for preserving both mother and fetus (35). Therefore, it is necessary to distinguish pregnant women with HFRS and other pregnancy-related pathologies. Moreover, the association of liver injury with different virus subtypes is another concern in our future study. In conclusion, it is necessary to weigh the influence of HFRS on mother when continuing the pregnancy against the likelihood of postnatal fetal survival.

Another concern relating to pregnant women with hantavirus infection is vertical transmission during pregnancy, as well as the subsequent impact on fetus. The positivity of viral markers in the spleen, liver, and lung tissue of stillborn fetuses support the putative vertical transmission described in earlier studies (30, 36, 37). Nonetheless, subsequent cases have not provided direct evidence of transplacental transmission (32, 38). In present study, no additional tests for markers of hantavirus were performed in newborns or placental tissue, and no clinical signs of HFRS were reported in newborns. Furthermore, as an acute infectious disease, positivity of viral markers in newborns is not a definite diagnostic marker, on account of the fact that these markers can be transmitted through the placenta. In our current investigation and that reported by Ji, there were cases involving the termination of pregnancy after recovery, largely due to concerns about the “potential influence” on fetuses. Nevertheless, considering the relatively favorable maternal and fetal outcomes observed in the mothers that continued pregnancy, termination is not recommended for patients who have recovered from HTNV infection.

## Limitations

This study has some inevitable limitations that need to be considered. Firstly, the retrospective nature of this study reduced the strength of the results and may have result in underestimation. However, prospective observational study or registry requires many years to reach a sample size that provides the statistical power to detect marked differences in the relevant risk compared with non-pregnant patients. Furthermore, there were only 27 pregnant patients with complete dataset; these were enrolled from one single medical center. The number of critical patients was relatively small, especially with regards to the binary logistic regression analysis used to identify risk factors for prognosis. Nevertheless, this study reported the largest number of cases in English literature, thus addressing the clear paucity of research on the clinical characteristics and pregnancy outcomes of patients with HFRS. Additionally, the diagnose of HFRS was based on serological positivity of IgM and/or IgG antibodies against hantavirus, subtypes of virus had not be detected. Herein, the association of clinical characteristics with different subtypes could not be analyzed in this retrospective cohort. A prospective controlled study will provide valuable information. Nevertheless, our data will help physicians to better understand and manage the pregnant women complicated with HFRS.

## Conclusion

Collectively, our findings indicate that pregnant women in the third trimester with HFRS have high likelihood of developing severe status; the fetuses of such patients are also at high risk of developing intrauterine adverse fetal events. We found that hypoalbuminemia was more common in pregnant women with HFRS and that these patients require comprehensive treatment to improve their pregnancy prognosis. Our results highlighted the predictive value of leukocytosis, especially a leukocyte count that was higher than 15 × 10^9^/L, for the early identification of severe cases; this will help us to monitor pregnant patients with HFRS. In addition, with timely diagnosis and comprehensive treatment, majority of the patients could undergo the remainder of the pregnancy. Therefore, elective termination after recovery is not suggested for vertical transmission or the so-called influence of hantavirus. Furthermore, public health education and vaccination against hantavirus should be implemented extensively, especially in women of childbearing age.

## Data Availability Statement

The original contributions presented in the study are included in the article/Supplementary Material, further inquiries can be directed to the corresponding authors.

## Ethics Statement

The studies involving human participants were reviewed and approved by The First Affiliated Hospital of Xi'an Jiaotong University with the approval of the Institutional Ethics Review Committee (No. XJTU1AF2021LSK-286). Written informed consent for participation was not required for this study in accordance with the national legislation and the institutional requirements.

## Author Contributions

All authors have contributed to and agreed on the content of the manuscript. DR, SF, and TY: study design, study identification, data collection and extraction, data analysis, interpretation, and manuscript drafting. TN and ZZ: data collection, quality assessment, and manuscript revision. MZ, JZ, and NY: data analysis and interpretation and critical revision of the manuscript. YY, YH, and TC: study design, data analysis, interpretation, and critical review of the manuscript. YZ and JL: study concept, study design, data analysis, interpretation of data, manuscript revision, quality control of algorithms, and study supervision.

## Funding

This research was supported by the Key R&D Program of Shaanxi (2018ZDXM-SF-037) and the Nature Science Foundation of Shaanxi (2020JM-394 and 2021JQ-404). The funders had no role in study design, data collection and analysis, decision to publish, or preparation of the manuscript.

## Conflict of Interest

The authors declare that the research was conducted in the absence of any commercial or financial relationships that could be construed as a potential conflict of interest.

## Publisher's Note

All claims expressed in this article are solely those of the authors and do not necessarily represent those of their affiliated organizations, or those of the publisher, the editors and the reviewers. Any product that may be evaluated in this article, or claim that may be made by its manufacturer, is not guaranteed or endorsed by the publisher.

## Abbreviations

HFRS: Hemorrhagic fever with renal syndrome
HPS: Hantavirus pulmonary syndrome
HTNV: Hantaan virus
SEOV: Seoul virus
MODS: Multiple organ dysfunction syndrome
DIC: Disseminated intravascular coagulation
ARDS: Acute respiratory distress syndrome
EMA: European Medicines Agency
CDC: Centers for Disease Control and Prevention
CRRT: Continuous renal replacement therapy
AFLP: Acute fatty liver of pregnancy
HELLP, Haemolysis: elevated liver enzymes and low platelet count.
