# Supplementary material for: The Clinical Characteristics and Outcomes of Hemorrhagic Fever With Renal Syndrome in Pregnancy
Source: Front Med (Lausanne). 2022 Feb 21;9:839224. doi: 10.3389/fmed.2022.839224 (PMC8899103; doi:10.3389/fmed.2022.839224)
Supplement: Supplementary file 1 [file Table_1.docx]

**Supplementary Table 1： Summary of included studies of HFRS Complicating Pregnancy**

| **Study** | **Country** | **No of cases** | **Mother's Age (year)** | **Gestation (week)** | **Virus species** | **Complications** | **Clinical classification of HFR** | **Delivery** | **Mother’s outcome** | **Fetal outcome** | **Reference** |
| --- | --- | --- | --- | --- | --- | --- | --- | --- | --- | --- | --- |
| Silberberg 1993 | France | 1 | 34 | 15 | Unknown | Unknown | Unknown | cesarean section | recovery | Survived and healthy | 12 |
| Howard 1999 | United States | 5 | 25 | 29 | SNV | ARDS;  pulmonary edema | Unknown | vaginal delivery. | recovery | Died; respiratory distress syndrome; cerebral palsy, seronegative | 39 |
|  |  |  | 34 | 13 | SNV | Unknown | Unknown | / | Died | Fetal death in utero; negative immunohistochemistry |  |
|  |  |  | 20 | 20 | SNV | ARDS | Unknown | vaginal delivery. | recovery | Survived and healthy |  |
|  |  |  | 27 | 17 | SNV | Respiratory failure | Unknown | vaginal delivery. | recovery | Survived and healthy |  |
|  |  |  | 28 | 16 | SNV | ARDS;  sepsis | Unknown | Unknown | recovery | Fetal death in utero |  |
| Duan  1997 | China | 48 | 21-34 | Early pregnancy (11/48); middle pregnancy (14/48); late pregnancy (23/48) | Unknown | Unknown | Mild (11);  Moderate (22); Severe (10);  Critical (5) | vaginal delivery (33/48) | Died (2/48); Recovered with  sequelae (19/42) | Survived (34); Premature birth (4;1 Survived;3 died); | 31 |
| Ma 2003 | China | 1 | 29 | 35 | HTNV | Acute renal failure;  Shock | Unknown | vaginal delivery. | recovery | Stillborn | 15 |
| Kim 2006 | Korea | 1 | 27 | 15 | HTNV | Shock | Unknown | vaginal delivery. | recovery | Survived and healthy | 14 |
| Schneider 2009 | France | 1 | 25 | 36 | PUUV | Unknown | Unknown | cesarean section | recovery | Survived but respiratory distress | 34 |
| Macé 2012 | France | 1 | mid-20s | 26 | SEOV | acute renal failure | Unknown | emergency Caesarean section | recovery | Premature birth and respiratory distress syndrome | 13 |
| Hofmann 2012 | Germany | 4 | 38 | 14 | DOBV | renal failure | Mild | vaginal delivery. | recovery | Survived and healthy | 33 |
|  |  |  | 23 | 28 | DOBV | acute renal failure | Mild | vaginal delivery. | recovery | Survived and healthy |  |
|  |  |  | 40 | 22 | PUUV | Unknown | Moderate | vaginal delivery. | recovery | Survived and healthy |  |
|  |  |  | 33 | 28 | PUUV | Unknown | Severe | cesarean section | recovery | Premature birth |  |
| Liu 2017 | China | 1 | 24 | 39 | Unknown | Shock | Unknown | cesarean section | recovery | Survived and healthy | 16 |
| Ji 2017 | China | 4 | 24 | 22 | HTNV | Pulmonary edema; severe hypoglycemia; encephalopathy | Critical | vaginal delivery. | recovery | Survived and healthy | 11 |
|  |  |  | 22 | 23 | HTNV | Pulmonary edema; hypoxemia | Severe | cesarean section | recovery | Survived and healthy |  |
|  |  |  | 28 | 13 | HTNV | NO | Moderate | vaginal delivery | recovery | Survived and healthy |  |
|  |  |  | 34 | 21 | HTNV | Pulmonary edema; bronchial pneumonia; visceral hemorrhage; hypoxemia; encephalopathy | Critical | Not available | recovery | Terminated of pregnancy |  |
| Qyra 2017 | Albania | 2 | 36 | 16.1 | HTNV | hypotensive | Unknown | Abortion | recovery | Abortion | 17 |
|  |  |  | 38 | 16.5 | HTNV | hypotensive | Unknown | Abortion | recovery | Abortion |  |
| Lu 2018 | China | 18 | / | 8-36 | Unknown | shock (12/18);  Bleedings (17/18) | Mild and Moderate (3/18);  Severe and Critical (15/18) | Unknown | recovery (14/18);  Died (4/18) | Unknown | 32 |
